# Analysis of a Methanogen and an Actinobacterium Dominating the Thermophilic Microbial Community of an Electromethanogenic Biocathode

**DOI:** 10.1155/2021/8865133

**Published:** 2021-03-01

**Authors:** Hajime Kobayashi, Ryohei Toyoda, Hiroyuki Miyamoto, Yasuhito Nakasugi, Yuki Momoi, Kohei Nakamura, Qian Fu, Haruo Maeda, Takashi Goda, Kozo Sato

**Affiliations:** ^1^Department of Systems Innovation, Graduate School of Engineering, The University of Tokyo, Tokyo 113-8656, Japan; ^2^Faculty of Applied Biological Sciences, Gifu University, Yanagido, Gifu 501-1193, Japan; ^3^Key Laboratory of Low-Grade Energy Utilization Technologies and Systems, Chongqing University, Ministry of Education, Chongqing 400044, China; ^4^INPEX Corporation, 9-23-30 Kitakarasuyama, Setagaya-ku, Tokyo 157-0061, Japan; ^5^Frontier Research Center for Energy and Resource (FRCER), Graduate School of Engineering, The University of Tokyo, Tokyo 113-8656, Japan

## Abstract

Electromethanogenesis refers to the bioelectrochemical synthesis of methane from CO_2_ by biocathodes. In an electromethanogenic system using thermophilic microorganisms, metagenomic analysis along with quantitative real-time polymerase chain reaction and fluorescence *in situ* hybridization revealed that the biocathode microbiota was dominated by the methanogen *Methanothermobacter* sp. strain EMTCatA1 and the actinobacterium *Coriobacteriaceae* sp. strain EMTCatB1. RNA sequencing was used to compare the transcriptome profiles of each strain at the methane-producing biocathodes with those in an open circuit and with the methanogenesis inhibitor 2-bromoethanesulfonate (BrES). For the methanogen, genes related to hydrogenotrophic methanogenesis were highly expressed in a manner similar to those observed under H_2_-limited conditions. For the actinobacterium, the expression profiles of genes encoding multiheme c-type cytochromes and membrane-bound oxidoreductases suggested that the actinobacterium directly takes up electrons from the electrode. In both strains, various stress-related genes were commonly induced in the open-circuit biocathodes and biocathodes with BrES. This study provides a molecular inventory of the dominant species of an electromethanogenic biocathode with functional insights and therefore represents the first multiomics characterization of an electromethanogenic biocathode.

## 1. Introduction

Electromethanogenesis refers to the bioelectrochemical synthesis of methane (CH_4_) from carbon dioxide (CO_2_) at the biocathodes of bioelectrochemical systems [1]. In such systems, catalytic microbes present on the cathode surface typically utilize electrons from the electrodes and reduce CO_2_. Because these biocathodes enable highly efficient conversion of electrical energy into methane, promising applications related to renewable electricity conversion (power to gas) and CO_2_ utilization have been proposed [2, 3].

Hydrogenotrophic methanogens, particularly those belonging to the family Methanobacteriaceae, appear to play a primary role in electromethanogenesis and are commonly detected as the dominant methanogen in biocathodes [1, 4, 5]. Recent studies of biocathodes inoculated with pure and cocultures revealed electron transfer pathways from the electrodes to methanogens. For example, direct electron uptake from negatively polarized electrodes was demonstrated in a methanogen of the family Methanobacteriaceae, namely, the iron-corroding *Methanobacterium*-like archaeon strain IM1 [6]. While some methanogens, including *Methanococcus maripaludis*, lack this ability [7], enzymes such as hydrogenases and the heterodisulfide reductase complex from *M. maripaludis* [7–9] adsorbed on the cathode surface have been shown to catalyze the production of soluble electron mediators such as H_2_ and formate using electrons from the electrodes, which can in turn be utilized by methanogens. Such mediators can also be produced by other microbes capable of direct electron uptake, such as the iron-corroding sulfate reducer *Desulfopila corrodens* strain IS4 [10, 11].

Despite the basic knowledge gained from defined culture systems, for practical applications, it is important to understand the mechanisms of electromethanogenesis in the multispecies microbial consortia enriched on biocathodes. Characterizing the functions of these constituent species is expected to lead to the identification of new catalysts, including methanogens and other species capable of electron uptake, as well as microbes with auxiliary functions (e.g., oxygen scavengers) and detrimental species (e.g., producers of undesirable products). These microbes may represent potential targets for the functional engineering of biocathodes for better performance and robustness. In two types of biocathodes, namely, a CO_2_-fixing aerobic biocathode and a biocathode primarily producing acetate, metagenomic analyses have revealed the compositions and metabolic capabilities of the surface microbial consortia [12–15]. In addition, active metabolic pathways, including those involved in CO_2_ fixation and electron transfer, and possible interspecies interactions have been inferred via metatranscriptomic and metaproteomic analyses [12, 15], providing crucial insights into the *in situ* functions of the various community members present at those biocathodes.

We previously reported the identification of a thermophilic microbial consortium that was capable of catalyzing electromethanogenesis at 55°C with a cathode poised at −0.35 V versus a standard hydrogen electrode (SHE) [16]. The results revealed that both methanogenesis and electron consumption at the biocathode were dependent on the presence of CO_2_ and were strongly inhibited by the methanogenesis inhibitor 2-bromoethanesulfonate (BrES). These findings suggested that the electrons from the cathode were primarily consumed for methanogenesis. Initial evaluation of relevant 16S rRNA clone libraries suggested that a methanogen related to *Methanothermobacter*, along with several other bacterial species, was enriched on the biocathode surface. Therefore, in this study, we aim to characterize the primary constituents of this consortium and intend to gain insight into their respective roles in the electromethanogenesis process.

## 2. Materials and Methods

### 2.1. Reactor Design and Operation

The specific characteristics and operating conditions of the reactors used in this study were generally the same as those described in our previous study [16]. Single-chamber reactors were constructed using 250 mL glass bottles. Two-chamber reactors comprising two identical 300 mL glass bottles separated by a pretreated proton exchange membrane (12.5 cm^2^, Nafion 117, DuPont Co., Wilmington, DE, USA) were also constructed. The anodes and cathodes were composed of plain carbon cloth (4 × 10 cm, TMIL Ltd., Ibaraki, Japan). Each electrode was connected to the circuit via a titanium wire (0.5 mm in diameter, Alfa Aesar, Ward Hill, MA, USA), which was directly fastened to the end of the electrode without glue. The internal resistance between the electrodes and titanium wires was less than 3.0 *Ω*. All reactors were sealed with butyl rubber stoppers and aluminum seals, and their headspaces were filled with N_2_/CO_2_ (80 : 20). The inoculated reactors were operated at 55°C in the fed-batch mode, in which the medium was exchanged with the fresh medium when current production was attenuated to the background level. A magnetic stirrer was continuously used in each chamber to provide sufficient mixing during the incubation.

#### 2.1.1. Operation of the Single-Chamber Reactor

The construction of active biocathodes was first initiated in the single-chamber reactor. The initial source of the microorganisms was the effluent of a preexisting bioelectrochemical reactor, which was originally inoculated with formation water from a petroleum reservoir [16]. Then, 25 mL of inoculum and 125 mL of sterile anaerobic medium containing 0.136 g/L of KH_2_PO_4_, 0.54 g/L of NH_4_Cl, 0.2 g/L of MgCl_2_·6H_2_O, 0.147 g/L of CaCl_2_·2H_2_O, 2.5 g/L of NaHCO_3_, and 10 mL/L of Wolfe's Mineral Solution supplemented with 0.8 g/L sodium acetate were added to the reactor. A constant voltage of 0.7 V was applied using a digital power supply (Array 3645A, Array Electronics, Nanjing, China). A fixed external resistance of 1.0 *Ω* was connected to the circuit. The voltage across the resistance was recorded every 5 min using a multimeter (34970A, Agilent Technologies, Santa Clara, CA, USA).

#### 2.1.2. Operation of the Two-Chamber Reactor

After three fed-batch cycles in the single-chamber reactor, the biocathode was gently rinsed using sterile anaerobic medium and then transferred to the cathode chamber of the two-chamber reactor for further analyses. In this setup, each anode and cathode chamber was filled with 200 mL of the anaerobic medium (with no sodium acetate). For the anode, a new abiotic electrode was used. An Ag/AgCl reference electrode (1 M KCl) with a potential of +0.20 V versus an SHE at 55°C was inserted into the cathode chamber. The biocathode, anode, and Ag/AgCl electrode were connected to a potentiostat (HSV-110, Hokuto Denko, Japan) as the working, counter, and reference electrodes, respectively. The biocathode was poised at a constant potential of −0.5 V versus an SHE. The reactor was operated in the fed-batch mode. In the study, the biocathodes were sacrificed for nucleic acid extraction and microscopic analyses after three fed-batch cycles in the two-chamber reactors.

### 2.2. Analytical Measurements

The gas composition in the reactor headspaces was analyzed using a gas chromatograph (GC-2014 equipped with a ShinCarbon ST column; Shimadzu, Kyoto, Japan) for each experiment. The pressure in the reactor headspace was measured using a digital pressure sensor (AP-C40; Keyence, Osaka, Japan). In the two-chamber reactor, cyclic voltammetry (CV) was performed using a standard three-electrode system. A potentiostat (HSV-110) was used in conjunction with the following parameters: equilibrium time of 99 s, scan rate of 1 Mv/s, and scanning range of −0.7 to −0.2 V versus an SHE.

### 2.3. Metagenome Analysis

Whole-genome shotgun sequencing of the biocathode-associated microbial community, assembling, and annotation of the metagenome have been described in previous reports [17, 18]. DNA was extracted from two independent biocathodes actively producing methane at −0.5 V versus an SHE using a DNeasy PowerMax Soil Kit (Qiagen, Hilden, Germany). The extracted DNA was sequenced in an Illumina HiSeq 2000 sequencer (150 bp paired-end sequencing). Adapter and quality trimming of the reads was performed using Cutadapt (version 1.8.3) [19]. The 16S rRNA gene amplicons of the biocathode-associated communities were sequenced using the extracted DNAs as the template, as previously described [20].

Approximately 395 million trimmed reads, approximately 60 gigabase pairs (Gbp), were used for the metagenomic binning. The MetaPhlAn2 tool (version 2.2.0) [21] was used to reveal the composition of the biocathode-associated consortium from the unassembled metagenomic reads. For assembling, reads were first downsampled to 400 megabase pairs (Mbp); as a result, sequences from relatively minor species were reduced. Then, the reads were assembled using the Velvet package (version 1.2.10) [22], followed by gap filling using the Sealer tool (ver.2.0.2) [23] and a quality check using the REAPR tool (version 1.0.18) [24]. The scaffolds were annotated using Prokka (version 1.13) [25]. For the metabolic pathway analysis, the proteins encoded in the draft genomes were mapped onto the Kyoto Encyclopedia of Genes and Genomes pathway database using the KEGG Mapper [26]. Mauve was used for alignment of linealized genomes [27].

### 2.4. Quantitative Real-Time Polymerase Chain Reaction (qPCR)

The shotgun-sequenced DNA and DNA samples extracted from other six independent biocathodes were used as templates in the qPCR analyses, which were performed in a LightCycler 480 system (Roche Diagnostics, Mannheim, Germany). Group-specific primers and probes designed by Yu et al. [28] were used for the methanogen of the order Methanobacteriales (i.e., *Methanothermobacter* sp. strain EMTCatA1), total archaea, and total bacteria (listed in Table S1). The primers and probes specific to the *Coriobacteriaceae* sp. strain EMTCatB1 were designed according to the 16S rRNA gene sequence of the strain and its three closely related sequences (FJ638596, KM819482, and AY753404) using the ARB program [29]. The DNA concentrations were quantified using a Qubit 3.0 fluorometer (Thermo Fisher Scientific) in conjunction with the Qubit dsDNA HS Assay Kit (Thermo Fisher Scientific). Each 10 *μ*L reaction mixture comprised 1 *μ*L of the template DNA, 300 nM of the specific primers, 200 nM of TaqMan probe, 5 *μ*L of Premix Ex Taq (TaKaRa, Kyoto, Japan), and PCR-grade sterilized water. PCR amplification was performed as follows: an initial 30 s of incubation at 95°C, 40 cycles of denaturation for 5 s each at 95°C, and annealing/extension for 30 s at 60°C. The amplification efficiency of the primer-probe sets was 1.80–1.86. Three separate trials were conducted for each sample. Standard curves for each assay were constructed using a synthetic 928 bp DNA fragment containing the target regions of the 16S rRNA genes of the *Methanothermobacter* sp. strain EMTCatA1 (for the archaeal and Methanobacteriales assays) and *Coriobacteriaceae* sp. strain EMTCatB1 (for the bacterial and *Coriobacteriaceae* species assays).

### 2.5. Microscopic Analyses

Fluorescence *in situ* hybridization (FISH) and scanning electron microscopy (SEM) were performed. FISH was used to identify *Methanothermobacter*-related methanogens using a probe specific to the 16S rRNA of the order Methanobacteriales (MB311, Table S1) [30]. To improve the penetration of the probe into the methanogens having pseudomurein cell walls, the FISH procedure was modified to include an enzymatic pretreatment of 4% (*w*/*v*) paraformaldehyde-fixed samples with recombinant pseudomurein endopeptidase (rPeiW), as previously described [31]. A probe specific to the 16S rRNA of the *Coriobacteriaceae* sp. strain EMTCatB1 (B1_648, Table S1) was designed using the ARB program [28]. The specificity and efficiency of the probe were preevaluated *in silico* using the SILVA TestProbe (version 3.0) [32] and the mathFISH tool [33]. For the permeabilization of gram-positive cell walls of the Actinobacteria, samples were fixed in 96% ethanol (without formaldehyde fixation) and pretreated with 10 mg/mL of lysozyme (Sigma-Aldrich, St. Louis, MO, USA) for 60 min, followed by digestion with 10 U/mL of achromopeptidase (Sigma-Aldrich) for 30 min before hybridization [34]. Both probes were labeled with Alexa Fluor 488 (Thermo Fisher Scientific, Waltham, MA, USA), and 5 *μ*M SYTO59 (Thermo Fisher Scientific) was used for counterstaining. The micrographs were analyzed using Fiji [35] to estimate the size distributions of the microbial cells on the cathode surfaces. For the SEM analysis, the electrodes were first fixed with 2.5% (*w*/*v*) glutaraldehyde and 2% (*w*/*v*) paraformaldehyde in 0.1 M phosphate buffer solution (PBS, pH 7.4) and processed as described previously [16].

### 2.6. RNA Extraction and Transcriptome Analysis

RNA samples were extracted from six independent biocathodes that produced methane actively at −0.5 V versus an SHE. In this experimental setup, biocathodes were clustered into two by establishing three experimental conditions, namely, closed-circuit (CC), open-circuit (OC), and BrES conditions. For this purpose, two biocathodes were directly subjected to RNA extraction (CC condition), whereas two other biocathodes were left in the open circuit for 5 h before RNA extraction (OC condition). For the next two biocathodes, BrES was anoxically injected into the cathode chambers at a final concentration of 12 mM, and the biocathodes were incubated with a poised potential of −0.5 V versus an SHE for 5 h before RNA extraction (BrES condition). Before being aseptically crushed, the biocathodes were soaked in LifeGuard Soil Preservation Solution (Qiagen) to stabilize the RNAs. Total RNA was then extracted using an RNeasy PowerSoil Total RNA Kit (Qiagen). Residual DNA was removed by DNase treatment using a TURBO DNA-free Kit (Thermo Fisher Scientific). From the total extracted RNA, mRNA was enriched by removing rRNA using a Ribo-Zero Kit (Illumina, San Diego, CA, USA). The enriched mRNA was then amplified using a MessageAmp II-Bacteria RNA Amplification Kit (Thermo Fisher Scientific) and further converted into cDNA using a SuperScript Double-Stranded cDNA Synthesis Kit (Thermo Fisher Scientific). The cDNA was then used to prepare a sequencing library using a TruSeq Stranded mRNA Library Prep Kit (Illumina). Metatranscriptome sequencing was performed by using an Illumina HiSeq 2500 system (Illumina), which yielded 100 bp pair-end reads totaling 51.7–108.3 million reads (Table S2). Eurofins Scientific Co. constructed the relevant libraries and performed all the sequencing reactions.

The RNA-seq reads were quality-filtered using Trimmomatic (version 0.36) [36] and aligned against the genomes of the *Methanothermobacter* sp. strain EMTCatA1 and *Coriobacteriaceae* sp. strain EMTCatB1 (AP018336 and BDLO01000001 in the DDBJ/EMBL/GenBank database) using BWA (version 0.7.17) [37]. The number of reads mapped onto the respective reference genomes was counted using SAMtools [38] and is shown in Table S3. Kaiju (version 1.7.2) [39] was used for the taxonomic assignment of the unmapped reads. StringTie (version 1.3.4d) [40] was used to assemble the mapped reads into transcripts and calculate the relative abundances of the assembled transcripts. Statistical analyses were performed using the edgeR package (version 3.16.1) [41]. Transcripts per million (TPM) was used to normalize the read counts to compare the expression levels across the genes for transcriptomes obtained from the same condition (Tables S4 and S5). Furthermore, the trimmed mean of *M*-values (TMM) was used to normalize the expression levels of each gene between the transcriptomes from the different conditions (Tables S6 and S7). The likelihood ratio test was used to determine the statistical significance of differences in gene expression. The Benjamini–Hochberg adjustment was used to control the false discovery rate (FDR) due to multiple hypothesis testing. For this, genes were considered to be differentially expressed if the expression level changed by more than two-fold (log2 fold change = 1) and the FDR was <0.05. Hierarchical clustering with average linkage was performed using the Pearson correlation dissimilarity metric, in which the cut-off distance, or dissimilarity, was 0.25.

## 3. Results and Discussion

### 3.1. Metagenomic Analysis of the Microbial Consortium of the Biocathodes

The biocathodes were subjected to metagenomic analysis to characterize the microbial composition and the metabolic potential of the surface microbial consortium. To minimize any potential effects due to sample variation, DNA was isolated from two independent biocathodes that were operated for more than 60 days with a poised potential of −0.5 V versus an SHE. Subsequent methane production rates (20.2 and 24.2 mmol CH_4_/day/cm^2^) and CV scans showed that the electromethanogenic activities of the two biocathodes were similar to each other (Figure S1A). Furthermore, 16S rRNA sequencing results suggested that the microbial compositions of the two biocathodes were also similar (Figure S1B). Thus, the DNA from the two biocathodes was combined at the library preparation step for whole-metagenome shotgun sequencing.

**Figure 1:**
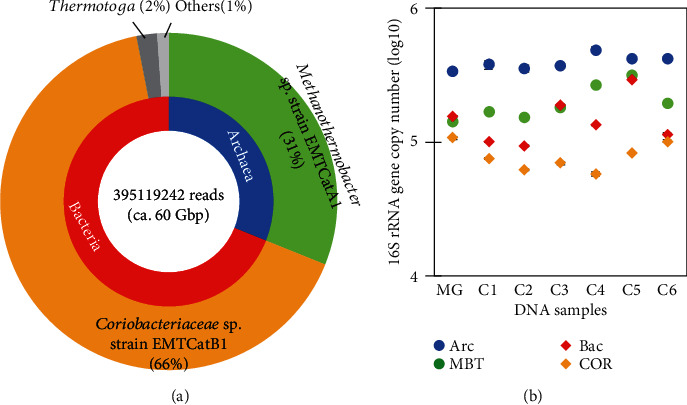
(a) Relative abundances and inferred taxonomies of the unassembled metagenome reads from the biocathode consortium. Kingdom-, genus-, and species-level identifications representing at least 1% relative abundance are shown. (b) The 16S rRNA gene copy numbers in the shotgun-sequenced DNA (MG) and the DNA samples extracted from the biocathodes (C1-C6). Copy numbers were quantified using primer-probe sets to detect the 16S rRNA genes of the domains Archaea (Arc) and bacteria (Bac), the order Methanobacteriales (MBT), and the *Coriobacteriaceae* sp. strain EMTCatB1 and related Actinobacteria (COR).

The assembly and binning of the Illumina sequencing reads indicated that the metagenome of the consortium was almost exclusively dominated by the sequences derived from the two dominant species (Figure 1(a)). Further assembling of the contigs, followed by gap filling, resulted in the reconstruction of the circular draft genomes of the two species (Figure S2) [17, 18]. Phylogenetic analyses of the marker genes demonstrated that one species was an archaeon that was closely related to methanogens of the genus *Methanothermobacter* (thus named *Methanothermobacter* sp. strain EMTCatA1). The genome of this species was identified to encode enzymes needed for hydrogenotrophic methanogenesis as well as a CO_2_ fixation pathway that proceeds via the incomplete reductive citrate cycle. The genome did not encode any apparent homolog of formate transporter or cytochrome. In particular, the *Methanothermobacter* sp. strain EMTCatA1 is closely related to the *M. thermautotrophicus* strain ∆H, a model organism of thermophilic methanogens, sharing 99% and 98% sequence identity of the 16S rRNA and *mcrA* genes, respectively. Moreover, the genome of the *Methanothermobacter* sp. strain EMTCatA1 is highly similar to the genome of the *M. thermautotrophicus* strain ∆H, sharing most of its genes with the *M. thermautotrophicus* strain ∆H in almost identical gene orders (Figure S3). Another species identified was a bacterium distantly related to Actinobacteria of the family Coriobacteriaceae (named *Coriobacteriaceae* sp. strain EMTCatB1) (Figure S4). The draft genome of the *Coriobacteriaceae* sp. strain EMTCatB1 was found to encode homologs of the enzymes required for anaerobic respiration (nitrite reduction), along with many putative redox proteins (e.g., 18 c-type cytochromes). These results were largely unexpected because 16S rRNA gene amplicon sequencing had suggested that although the *Methanothermobacter*-related methanogen was shown to be a primary archaeal constituent, the bacterial population was composed of more diverse bacteria (Figure S1B). Indeed, in the 16S rRNA gene amplicons, actinobacterial species represented only a relatively minor proportion of the sequences (Figure S1B). Underestimation of actinobacterial species in 16S rRNA gene amplicons has previously been reported and is presumably due to the high GC content of these genomes [42, 43]. For example, the GC content is 67.2% in the case of the *Coriobacteriaceae* sp. strain EMTCatB1.

### 3.2. qPCR Analysis of the Biocathode Microbial Consortia

To examine the abundances of these two species on the biocathode surfaces, 16S rRNA gene copy numbers were estimated using qPCR, along with group-specific primers (Figure 1(b)). In addition to the shotgun-sequenced DNA (MG in Figure 1(b)), DNA samples were extracted from biocathodes of six independent electromethanogenic reactors (named C1~C6) and analyzed (C1-C6 in Figure 1(b)). CV confirmed the ability of the six biocathodes to catalyze electrochemical reactions (Figure S5), which is consistent with previously reported results [16]. The surface microbial colonization was confirmed by SEM (Figure S6). In 20 pg of DNA, the copy numbers of archaeal 16S rRNA genes ranged from 3.4 ± 0.05 × 10^5^ to 4.9 ± 0.2 × 10^5^. The copy numbers of the 16S rRNA genes of the order Methanobacteriales (including the genus *Methanothermobacter*) ranged from 1.4 ± 0.02 × 10^5^ to 3.1 ± 0.7 × 10^5^, which corresponded to an average of 51% of the copy numbers of the total archaeal 16S rRNA genes. Bacterial 16S rRNA gene copy numbers ranged from 9.3 ± 0.07 × 10^4^ to 2.9 ± 0.03 × 10^5^. For the *Coriobacteriaceae*-related species, gene copy numbers ranged from 5.8 ± 0.1 × 10^4^ to 1.1 ± 0.02 × 10^5^, which corresponded to an average of 52% of the total bacterial 16S rRNA gene copy numbers. It should be noted that the primers of the 16S rRNA genes likely overestimated the abundance of Methanobacteriales, and therefore, *Archaea*, as the draft genome of the *Methanothermobacter* sp. strain EMTCatA1, contained two copies of the 16S rRNA gene. Nonetheless, absolute quantification of the 16S rRNA gene copy numbers supported the dominance of the two species on the biocathode surfaces.

### 3.3. FISH Analysis of Microbial Cells on the Biocathode Surfaces

**Figure 2:**
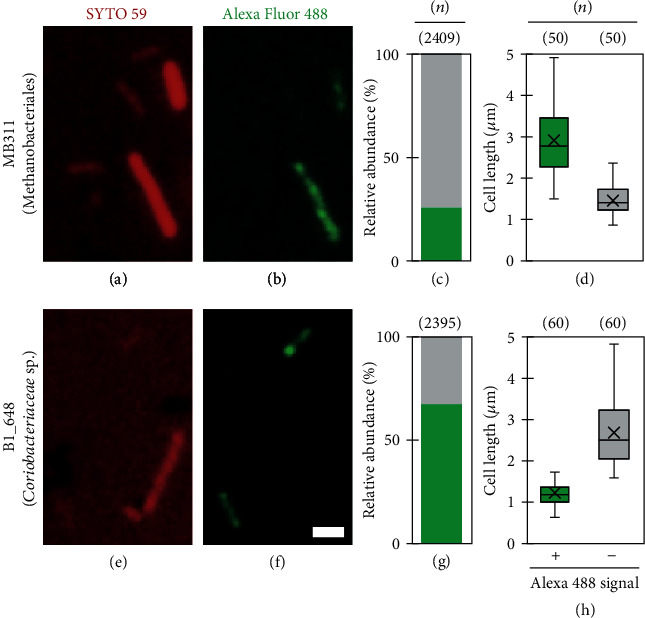
FISH analysis of the microbial populations present on the cathode surfaces and in the supernatants of the cathode chambers. Alexa Fluor 488-labeled probes targeting the order Methanobacteriales (MB311) and *Coriobacteriaceae* sp. strain EMTCatB1 (B1_648) were used. SYTO59 was used for counterstaining. (a, b, e, f) Epifluorescent micrographs of two representative fields separately capturing the fluorescence of Alexa Fluor 488 (green) and SYTO59 (red) using the probes MB311 and B1_648, respectively (scale bar: 1.0 *μ*m). (c, g) Stacked bar charts (100%) of the relative abundances of the cells with the Alexa Fluor 488 signal (green-colored stacks) and without the signal (gray-colored stacks) on the cathode surfaces. (d, h) Box and whisker plots of the cell lengths with the Alexa Fluor 488 signal (+) and without the signal (−). The number of counted/measured cells (*n*) is indicated at the top of the panels (c), (d), (g), and (h).

FISH analysis further confirmed that the two species represented the major constituents of the microbial populations on the biocathode surfaces (Figure 2). The epifluorescence micrographs showed that approximately 26% of the microbes were labeled with the probe for methanogens of the family Methanobacteriales, thereby targeting the strain EMTCatA1 (Figures 2(a)–2(c)), and 68% of the cathode-associated microbes were labeled with the probe targeting the *Coriobacteriaceae* sp. strain EMTCatB1 (Figures 2(e)–2(g)). In particular, the cells labeled with probes targeting EMTCatA1 were relatively longer filamentous cells or rods, with a median length of 2.8 *μ*m, compared with the unlabeled cells, which had a median length of 1.5 *μ*m (Figure 2(d)). This finding was consistent with the previous reports of other *Methanothermobacter* species [44, 45]. In contrast, the cells labeled with probes targeting the EMTCatB1-labeled rod cells having a median length of 1.2 *μ*m were mostly shorter than the unlabeled cells, which had a median length of 2.5 *μ*m (Figure 2(h)). Therefore, it can be concluded that the surface communities of the biocathodes were primarily composed of two types of cells: long rods or filamentous cells (typically longer than 1.6 *μ*m) of the *Methanothermobacter* sp. strain EMTCatA1 and relatively short rods (typically shorter than 1.6 *μ*m) of the *Coriobacteriaceae* sp. strain EMTCatB1 (Figures 2(d) and 2(h)).

### 3.4. Transcriptome Analysis of the Dominant Species on the Biocathode Surfaces

Metatranscriptomes of the biocathodes under the CC, OC, and BrES conditions were analyzed to gain insight into the respective roles of the dominant species in electromethanogenesis. As we previously observed [16], methanogenesis ceased at the biocathodes under the OC condition, in addition to the BrES condition, in which both methanogenesis and electron consumption processes at the biocathode were inhibited (Figure S7). For all the metatranscriptomes, 69%–92% of the reads were mapped onto the genomes of the two species (Table S2), further indicating that they were the main metabolically active species at the biocathode. The unmapped reads were assigned to diverse taxa (Figure S8). No taxon appeared to be commonly overrepresented among the unmapped reads. Therefore, as this study focused on the dominant species, the unmapped reads were excluded from further analyses.

The transcriptome profiles under the CC condition were analyzed, with a particular focus on highly transcribed genes related to energy metabolism and electron transfer to identify the candidate genes involved in electromethanogenesis. Hierarchical clustering of the differentially expressed genes (significance criteria: FDR < 0.05, fold change > 2) among the conditions was used to estimate the influence of the electron supply from the cathode and the methanogenic activity on the physiology of the dominant species.

#### 3.4.1. Transcriptome Analysis of the *Methanothermobacter sp.* Strain EMTCatA

**Figure 3:**
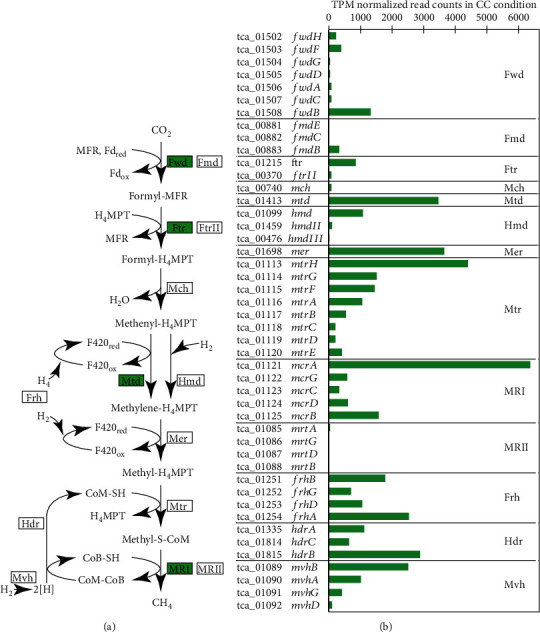
The methanogenesis pathway and gene expression patterns of methanogenesis-related genes of the *Methanothermobacter* sp. strain EMTCatA1. (a) Enzymes catalyzing respective reactions in the pathway are indicated in boxes. Fwd: tungsten-containing formyl-MFR dehydrogenase; Fmd: molybdenum-containing formyl-MFR dehydrogenase; Ftr: formyl-MFR:H_4_MPT formyltransferase; Mch: N_5_N_10_-methenyl-H_4_MPT cyclohydrolase; Mtd: F_420_-dependent N_5_N_10_-methylene-H_4_MPT dehydrogenase; Hmd: H_2_-dependent N_5_N_10_-methylene-H_4_MPT dehydrogenase; Mer: F_420_-dependent N_5_N_10_-methylene-H_4_MPT reductase; Mtr: N_5_-methyl-H_4_MPT methyltransferase; MRI: methyl-CoM reductase I; MRII: methyl-CoM reductase II; Hdr: heterodisulfide reductase; Mvh: methyl viologen-reducing hydrogenase; Frh: F_420_-reducing hydrogenase; MFR: methanofuran; Fd: ferredoxin; H4MPT: tetrahydromethanopterin; CoM-SH: coenzyme M; CoB-SH: coenzyme B. Isofunctional enzymes expressed at higher levels are highlighted in green. (b) TPM-normalized read counts of methanogenesis-related genes in CC condition.

For the *Methanothermobacter* sp. strain EMTCatA1, the mRNAs related to hydrogenotrophic methanogenesis were among the most highly abundant under the CC condition, with 15 of 46 methanogenesis-related genes in the top 10% of abundant transcripts (Table S4). Notably, *mcrA* (tca_01121), *mtd* (tca_01413), and *mer* (tca_01698), which encoded the homologs of a subunit of methyl-coenzyme M reductase I (MRI) and two cofactor F_420_-dependent enzymes, respectively, were found to be expressed to a greater extent than the genes for their isofunctional enzymes (e.g., MRII and H_2_-dependent enzymes) (Figure 3). In closely related *M. thermautotrophicus* strains, the expression of MRI and enzymes involved in cofactor F_420_-dependent reactions was induced under H_2_-limited conditions (e.g., syntrophic cocultures) [46–54].

**Figure 4:**
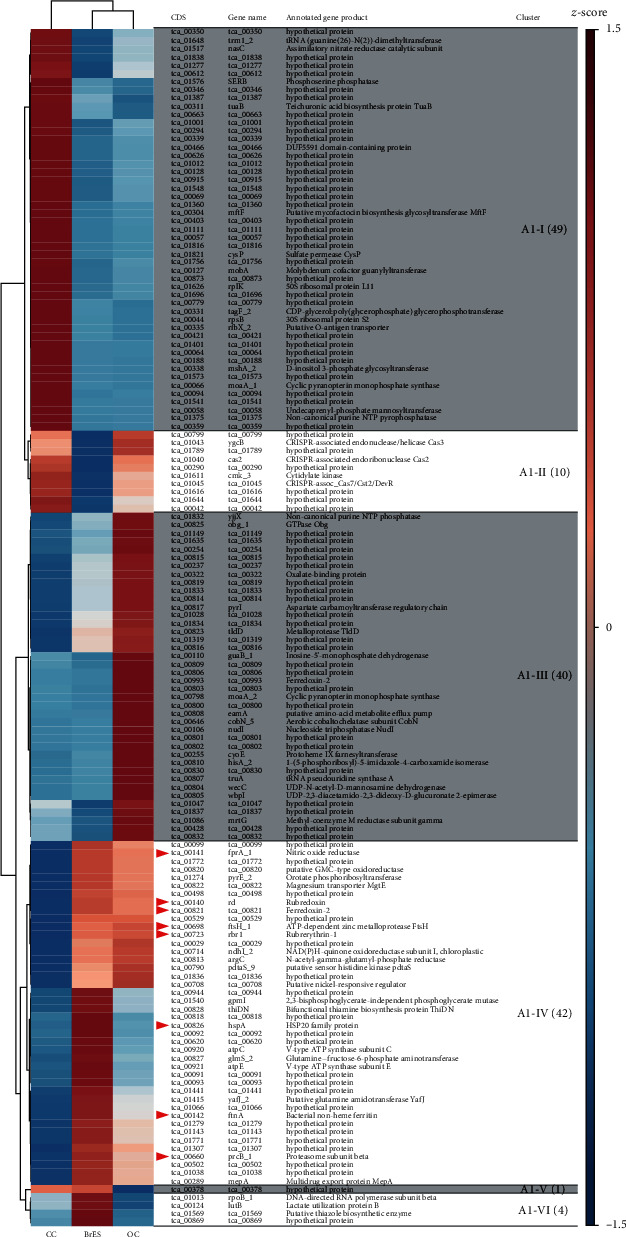
Heatmap and hierarchical clustering of the differentially expressed genes of the *Methanothermobacter* sp. strain EMTCatA1. The scale of the heatmaps was the average of the TMM-normalized count values transformed so that the mean was 0 and the standard deviation was 1 (*z*-score). The assigned clusters were indicated at the rightmost column, with the number of genes contained in each cluster shown in brackets. The red arrowheads indicate the stress-related genes.

Among the transcriptomes of the *Methanothermobacter* sp. strain EMTCatA1, 146 genes were found to be differentially expressed (Table S6). Based on the hierarchical clustering of the differential expression patterns, six clusters of the differentially expressed genes were identified (Figure 4, Table S8). The largest cluster (A1-I) consisted of 49 genes that were expressed at higher levels under the CC condition than at those under the BrES or OC conditions. Overall, 28 genes in the A1-I cluster encoded hypothetical proteins of unknown functions. A1-IV, the second-largest cluster, consisted of 42 genes that were expressed at higher levels under both the BrES and OC conditions than under the CC condition. Eight genes in the A1-IV cluster encoded homologs of various stress-related proteins, such as chaperones and proteasomes (tca_00660, tca_00698, and tca_00826), antioxidant enzymes, and alternative redox proteins (tca_00140, tca_00141, tca_00142, tca_00723, and tca_00821) (marked by red arrowheads in Figure 4) (Table S8). This likely reflected the process of cellular energy depletion due to the lack of methanogenesis under the OC and BrES conditions. No gene encoding an apparent homolog related to direct electron uptake was identified.

Although methanogenesis ceased under the OC and BrES conditions, methanogenesis-related genes did not match our criteria for differential expression. For other methanogens, the expression of methanogenesis genes was reported to be controlled by H_2_ availability and was not significantly affected by treatments that inhibited methanogenesis [46, 55]. This was also consistent with the vital role of methanogenesis in methanogens. An exception was *mrtG* (tca_01086), which encoded an MRI gamma-subunit homolog. Although this gene demonstrated higher expression under the OC condition than under the other conditions (in cluster A1-III in Figure 4), the associated transcript abundances were barely detectable and, therefore, unlikely to have biological significance (Table S8).

#### 3.4.2. Transcriptome Analysis of the *Coriobacteriaceae* sp. Strain EMTCatB1

For the *Coriobacteriaceae* sp. strain EMTCatB1, genes encoding the putative multiheme c-type cytochromes (1d0125, 1c0363, 1c0406, 1d0898, and 1c0642), a NiFe hydrogenase (1c0061, 1c0060, and 1c0059), and a formate dehydrogenase (1c0408 and 1c0407 with the abovementioned 1c0406) were found to be highly expressed under the CC conditions (in the top 10% abundant transcripts: Tables S5 and S9). Both multiheme c-type cytochromes and membrane-bound oxidoreductases have been proposed to constitute an extracellular electron transfer conduit in *Thermincola potens*, an exoelectrogenic gram-positive bacterium [56, 57]. In addition, a gene cluster encoding the subunits of a V-type ATPase (1d0031-37, Table S5) was highly transcribed.

**Figure 5:**
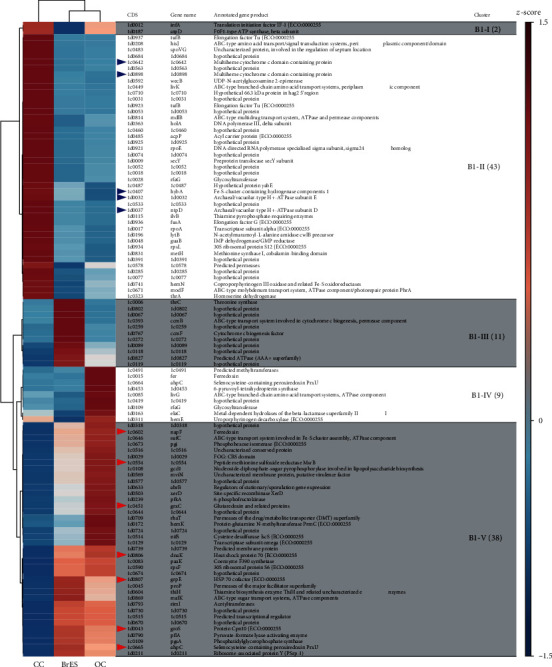
Heatmap and hierarchical clustering of the differentially expressed genes of the *Coriobacteriaceae* sp. strain EMTCatB1. The scale of the heatmaps was the average of the TMM-normalized count values transformed so that the mean was 0 and the standard deviation was 1 (*z*-score). The assigned clusters were indicated at the rightmost column, with the number of genes contained in each cluster shown in brackets. The blue arrowheads indicate the genes potentially involved in cathodic electron consumption. The red arrowheads indicate the stress-related genes.

Among the transcriptomes of the *Coriobacteriaceae* sp. strain EMTCatB1, 103 differentially expressed genes were identified (Figure 5, Table S7). The general cluster organization of the differentially expressed genes was similar to that in the *Methanothermobacter* sp. strain EMTCatA1 (Figure 5, Table S10). The largest cluster (B1-II), which consisted of 43 genes, was expressed at higher levels under the CC condition, and conversely, the second-largest cluster (B1-V), which consisted of 38 genes, was expressed at higher levels under the BrES and OC conditions. Cluster B1-II contained the abovementioned genes encoding two multiheme c-type cytochromes (1d0898 and 1c0642), a hydrogenase component of formate dehydrogenase (1c0407), and two subunits of V-type ATPase (1d0032 and 1d0037) (blue arrowheads in Figure 5) (Table S10). These results suggest that these genes play a role in electron consumption at the biocathode surfaces. Furthermore, similar to the A1-IV cluster of the strain EMTCatA1, the B1-V cluster consisted of stress-related genes encoding homologs of chaperones (1d0043, 1d0806, and 1d0807), antioxidant enzymes (1c0451, 1c0554, and 1c0665), and an alternative redox protein (1c0602) (red arrowheads in Figure 5) (Table S10), suggesting that the bacterium was under stress in both the BrES and OC conditions.

### 3.5. Possible Metabolic Functions of the Dominant Species on the Biocathode Surface

Based on our present study results and those from our previous study [16], we investigated the possible roles of the dominant species in the electromethanogenesis process. We concluded that the *Methanothermobacter* sp. strain EMTCatA1 was responsible for methanogenesis at the biocathodes via the hydrogenotrophic methanogenesis pathway, which was operated in a manner similar to that under H_2_-limited conditions. It is possible that this methanogen alone catalyzed electromethanogenesis via direct electron uptake from the cathode, a phenomenon that was previously reported for the *Methanobacterium*-like archaeon strain IM1 [6] and various *Methanosarcina* species [58]. However, this might not be the case for the *Methanothermobacter* sp. strain EMTCatA1 as the pure cultures of the *M. thermautotrophicus* strain ∆H, a close relative of the *Methanothermobacter* sp. strain EMTCatA1, demonstrated no catalytic ability on a cathode poised at a potential higher than −0.6 V versus an SHE [16]. Only 14 genes of the *Methanothermobacter* sp. strain EMTCatA1, including two CRISPR-associated genes, namely, tca_01044 and tca_01045, had no apparent homolog in the *M. thermautotrophicus* strain ∆H (Table S11). Although some of these genes might confer the capability for direct electron uptake in the methanogen, we presumed that the *Methanothermobacter* sp. strain EMTCatA1 was a hydrogenotrophic methanogen highly similar to the *M. thermautotrophicus* strain ∆H and was likely unable to catalyze electromethanogenesis by itself.

The role of the *Coriobacteriaceae* sp. strain EMTCatB1 in electromethanogenesis remained more speculative. Based on its gene expression profile, it is presumable that the *Coriobacteriaceae* sp. strain EMTCatB1 was capable of direct electron uptake from the cathode via multiheme c-type cytochromes (e.g., those encoded by 1d0898 and 1c0642). The electrons were then likely conducted to the relevant membrane-bound oxidoreductases, such as hydrogenase and formate dehydrogenase. At the least, part of the electrical energy from this electron flow was possibly utilized to create a proton motive force to drive ATP synthesis via V-type ATPase. In addition, NADH:ubiquinone oxidoreductase (complex I) might be involved in the generation of a proton motive force as two genes encoding subunits of the enzyme (*nuoCD*: 1c0337 and 1c0336) were highly transcribed under the CC condition (Table S5). Therefore, cellular energy was likely depleted under the OC condition, which was consistent with the induction of stress-related genes.

### 3.6. A Possible Model of the Electromethanogenesis Process on the Biocathode Surface

Based on our results and discussions, it is tempting to speculate that a significant proportion of the electrons from the cathode was channeled to proton reduction in the *Coriobacteriaceae* sp. strain EMTCatB1, resulting in H_2_ evolution. The *Methanothermobacter* sp. strain EMTCatA1 then consumed the released H_2_ for hydrogenotrophic methanogenesis. In the *Coriobacteriaceae* sp. strain EMTCatB1, higher expression of stress-related genes was observed under the BrES condition, suggesting that the addition of BrES affected the physiology of the bacterium. This observation could be interpreted that the bacterium needed methanogenesis for its metabolic activity on the cathode. In other words, hydrogenotrophic methanogenesis by the methanogen served to reduce the H_2_ partial pressure. Therefore, it kept its metabolism thermodynamically favorable. Thus, the two dominant species might be metabolically interdependent at the biocathode surface, performing obligately mutualistic metabolism [59] that serves to catalyze electromethanogenesis. Bacteria related to the family Coriobacteriaceae have been detected as the dominant species in another biocathode [60], supporting the notion that Actinobacteria play a role in electromethanogenesis.

However, the above model was highly speculative and required future investigations. A considerable limitation was the current lack of physiological knowledge regarding the dominant species. In particular, the *Coriobacteriaceae* sp. strain EMTCatB1 had no close relatives among the cultured species (Figure S4), and its metabolic properties remained mostly unknown. The response of the *Coriobacteriaceae* sp. strain EMTCatB1 to BrES could well be due to the direct effect; i.e., BrES was toxic to or metabolized by the bacterium, thereby altering its gene expression pattern. Moreover, as the genome does not encode conserved enzymes for CO_2_ fixation, it is unclear how the bacterium grows on the cathode. In this regard, it is plausible that the *Coriobacteriaceae* sp. strain EMTCatB1 utilized acetate as its carbon source, which was present in the medium only during the initial development of the biocathode (in the single-chamber reactor) and then omitted from the medium (in the two-chamber reactor). In other words, the bacterium might not be able to propagate on the biocathode (in the absence of acetate) but still metabolically active and able to produce H_2_.

Therefore, future studies should perform isolation of the dominant species, together with biochemical analyses [56, 57, 61]. Such an approach would also be useful to examine the possible contribution of relatively minor species, which were excluded from this study, to elaborate the electromethanogenesis process. Further transcriptome/proteome analyses using different conditions, such as using various electrical potential values, are also required to understand the biocathode mechanism more comprehensively.

## 4. Conclusions

The primary constituents of a novel thermophilic consortium enriched on an electromethanogenic biocathode were characterized in the present study. The results indicated that the metagenome of the consortium was mainly dominated by the *Methanothermobacter* sp. strain EMTCatA1 and *Coriobacteriaceae* sp. strain EMTCatB1. The dominance of the two species on the biocathodes was further confirmed using qPCR and FISH, leading us to analyze the transcriptomes in the biocathodes under different conditions (CC, OC, and BrES). Based on the expression profile of the genes involved in hydrogenotrophic methanogenesis by the methanogen and those encoding c-type cytochromes and membrane-bound enzymes of the bacterium, these strains were suggested to have functions in methane production and electron uptake, respectively, in the electromethanogenesis process. This study therefore represents the first multiomics characterization of an electromethanogenic biocathode, providing complementary information for previous studies on various bioelectrochemical systems.

## Data Availability

Raw reads of the metagenome, metatranscriptomes, and 16S rRNA gene amplicons were deposited in the Sequence Read Archive of the DNA Data Bank of Japan (DDBJ) under the BioProjects PRJDB8994, PRJDB8993, and PRJDB8998, respectively.

## Supplementary Materials

Supplementary Materials Supplementary figures (characterization of the metagenome-sequenced biocathodes (Figure S1), genome maps of the two dominant species (Figure S2), whole genome alignment of the *Methanothermobacter* sp. strain EMTCatA1 and *M. thermautotrophicus* strain ∆H (Figure S3), phylogenetic tree of the *Coriobacteriaceae* sp. strain EMTCatB1 and other Actinobacteria (Figure S4), cyclic voltammograms of the biocathodes of the electromethanogenic reactors C1~C6 (Figure S5), representative scanning electron micrographs of the biocathode surfaces of the electromethanogenic reactors C1~C6 (Figure S6), current generation and CH_4_ production profiles of the biocathodes used for the transcriptome analysis (Figure S7), and taxonomic assignments of unmapped RNA-seq read (Figure S8)) and tables (details on probes and primers (Table S1), RNA sequencing and mapping (Tables S2 and S3), TPM- (Tables S4 and S5) and TMM- (Tables S6 and S7) normalized read counts and differentially expressed gene clusters (Tables S8 and S10) of the transcriptomes of the dominant species, c-type cytochromes of the *Coriobacteriaceae* sp. strain EMTCatB1 (Table S9), and genes of the *Methanothermobacter* sp. strain EMTCatA1 not present in the *M. thermautotrophicus* strain ∆H (Table S11)) are available online.
