# Supplementary material for: Analysis of a Methanogen and an Actinobacterium Dominating the Thermophilic Microbial Community of an Electromethanogenic Biocathode
Source: Archaea. 2021 Mar 1;2021:8865133. doi: 10.1155/2021/8865133 (PMC7943316; doi:10.1155/2021/8865133)
Supplement: Supplementary Materials — Supplementary figures (characterization of the metagenome-sequenced biocathodes (Figure S1), genome maps of the two dominant species (Figure S2), whole genome alignment of the Methanothermobacter sp. strain EMTCatA1 and M. thermautotrophicus strain ∆H (Figure S3), phylogenetic tree of the Coriobacteriaceae sp. strain EMTCatB1 and other Actinobacteria (Figure S4), cyclic voltammograms of the biocathodes of the electromethanogenic reactors C1~C6 (Figure S5), representative scanning electron micrographs of the biocathode surfaces of the electromethanogenic reactors C1~C6 (Figure S6), current generation and CH4 production profiles of the biocathodes used for the transcriptome analysis (Figure S7), and taxonomic assignments of unmapped RNA-seq read (Figure S8)) and tables (details on probes and primers (Table S1), RNA sequencing and mapping (Tables S2 and S3), TPM- (Tables S4 and S5) and TMM- (Tables S6 and S7) normalized read counts and differentially expressed gene clusters (Tables S8 and S10) of the transcriptomes of the dominant species, c-type cytochromes of the Coriobacteriaceae sp. strain EMTCatB1 (Table S9), and genes of the Methanothermobacter sp. strain EMTCatA1 not present in the M. thermautotrophicus strain ∆H (Table S11)) are available online. [file 8865133.f1.zip › Supporting_Information_Kobayashi_et_al/Supporting_Information_Kobayashi_et_al.docx]

**Supporting Information for**

**Analysis of a Methanogen and an Actinobacterium Dominating the Thermophilic Microbial Community of an Electromethanogenic Biocathode**

Hajime Kobayashi^a,b,*^, Ryohei Toyoda^a^, Hiroyuki Miyamoto^a^, Yasuhito Nakasugi^a^, Yuki Momoi^a^, Kohei Nakamura^c^, Qian Fu^d^, Haruo Maeda^e,†^, Takashi Goda^a^, and Kozo Sato^a,b^

^a^*Department of Systems Innovation, Graduate School of Engineering, The University of Tokyo, Tokyo 113-8656, Japan*

^b^*Frontier Research Center for Energy and Resource (FRCER), Graduate School of Engineering, The University of Tokyo, Tokyo 113-8656, Japan*

^c^*Faculty of Applied Biological Sciences, Gifu University, Yanagido, Gifu 501-1193, Japan*

^d^*Key Laboratory of Low-Grade Energy Utilization Technologies and Systems, Chongqing University, Ministry of Education, Chongqing 400044, China*

^e^*INPEX Corporation, 9-23-30 Kitakarasuyama, Setagaya-ku, Tokyo 157-0061, Japan*

^†^Present affiliation: Haruo Maeda, Institute for Geo-Resources and Environment, Geological Survey of Japan, National Institute of Advanced Industrial Science and Technology (AIST), 1-1-1 Higashi, Tsukuba 305-8567, Japan

^*^Corresponding author: Hajime Kobayashi, Department of Systems Innovation, Graduate School of Engineering, The University of Tokyo, Eng. Bldg. No. 3, 7-3-1 Hongo, Bunkyo-ku, Tokyo 113-8656, Japan. Phone: +81-(3)-5841-7054; Fax: +81-(3)-3818-7492. E-mail: [kobayashi@frcer.t.u-tokyo.ac.jp](mailto:kobayashi@frcer.t.u-tokyo.ac.jp)

**Number of pages: 10**

**Contents**

**Supplementary figures (number of figures: 8)**

**Figure S1:** Characterization of two biocathodes (MG1 and MG2) from which DNA was extracted for metagenome analysis.

**Figure S2:** Schematic representation of the reconstructed draft genomes of *Methanothermobacter* sp. strain EMTCatA1 and *Coriobacteriaceae* sp. strain EMTCatB1.

**Figure S3:** Whole genome alignment of *Methanothermobacter* sp. strain EMTCatA1 and *M. thermautotrophicus* strain ∆H.

**Figure S4:** Phylogenetic tree illustrating relationships between *Coriobacteriaceae* sp. strain EMTCatB1 and other actinobacteria species.

**Figure S5:** Cyclic voltammograms of the biocathodes of the electromethanogenic reactors C1 ~ C6.

**Figure S6:** Representative scanning-electron micrographs of the biocathode surfaces of the electromethanogenic reactors C1 ~ C6.

**Figure S7:** Current generation and CH_4_ production by the biocathodes used for the transcriptome analysis.

**Figure S8:** Taxonomic assignment of unassembled RNA-seq reads, which were not mapped onto *Methanothermobacter* sp. strain EMTCatA1 and *Coriobacteriaceae* sp. strain EMTCatB1 genomes, in metatranscriptome samples.

**Supplementary Tables (Number of tables: 11) (in attached excel file “Supplemental_Tables.xlsx”)**

**Table S1.** Primers and probes used in this study.

**Table S2.** Overview of RNA sequencing

**Table S3.** Mapping of RNA-seq reads onto the genome of the dominant species

**Table S4.** TPM normalized read counts of *Methanothermobacter* sp. strain EMTCatA1 transcriptomes sorted by expression levels under the CC condition.

**Table S5.** TPM normalized read counts of *Coriobacteriaceae* sp. strain EMTCatB1 transcriptomes sorted by expression levels under the CC condition.

**Table S6.** TMM normalized read counts of *Methanothermobacter* sp. strain EMTCatA1 transcriptomes sorted by FDR

**Table S7.** TMM normalized read counts of *Coriobacteriaceae* sp. strain EMTCatB1 transcriptomes sorted by FDR.

**Table S8.** Clusters of differentially expressed genes of *Methanothermobacter* sp. strain EMTCatA1 based on hierarchical clustering of differential expression patterns.

**Table S9.** Genes of *Coriobacteriaceae* sp. strain EMTCatB1 encoding putative multiheme c-type cytochromes.

**Table S10.** Clusters of differentially expressed genes of *Coriobacteriaceae* sp. strain EMTCatB1 based on hierarchical clustering of differential expression patterns.

**Table S11.** Genes of *Methanothermobacter* sp. strain EMTCatA1 that have no homolog in *M. thermautotrophicus* strain ∆H.


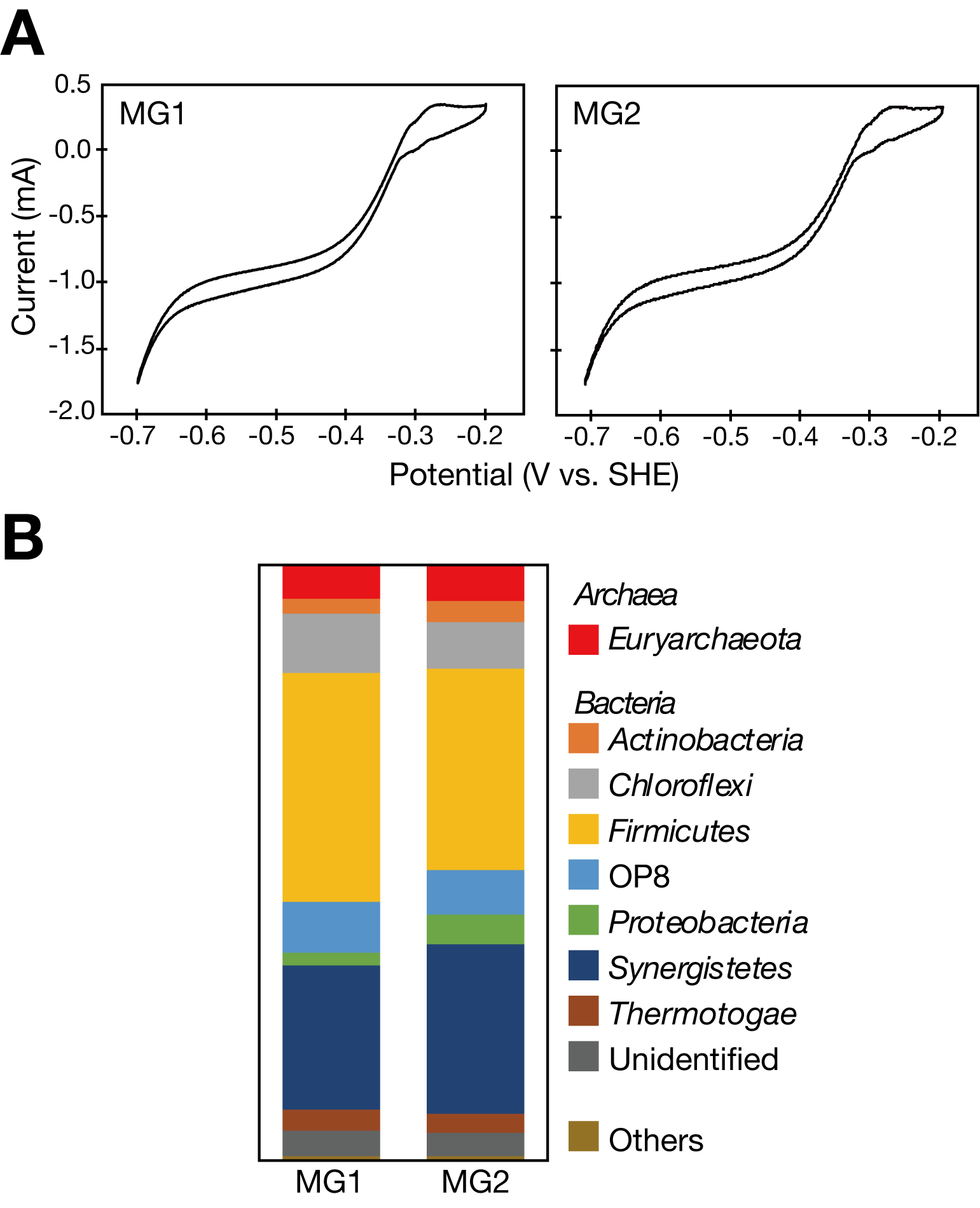


**FIGURE S1:** Characterization of two biocathodes (MG1 and MG2) from which DNA was extracted for metagenome analysis. (A) Cyclic voltammograms of the two biocathodes. (B) Microbial diversity of the biocathode-associated communities based on 16S rRNA gene amplicon sequencing. Relative abundance of major taxonomic groups (phylum level) are shown. Taxa representing <1% of sequences are grouped in the ‘Others’ category.


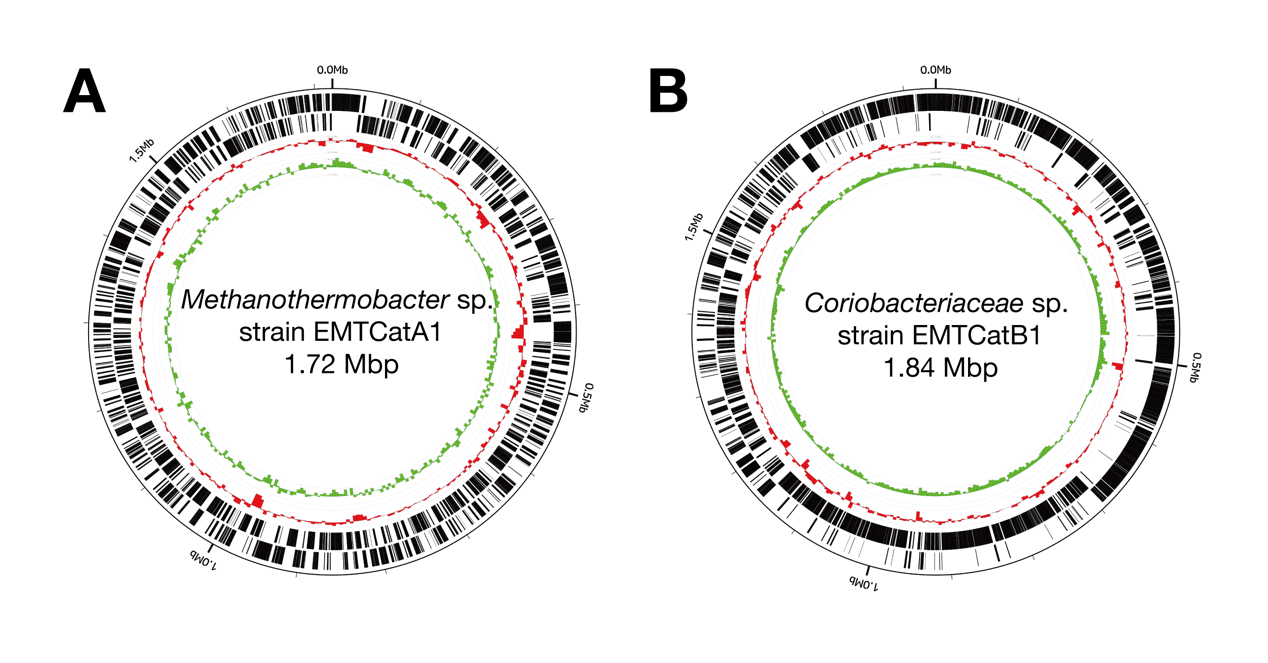


**FIGURE S2:** Schematic representation of the reconstructed draft genomes of (A) *Methanothermobacter* sp. strain EMTCatA1 and (B) *Coriobacteriaceae* sp. strain EMTCatB1 showing coordinate labels, forward and reverse strand genes, percent GC content, and GC-skew (from outside to inside).


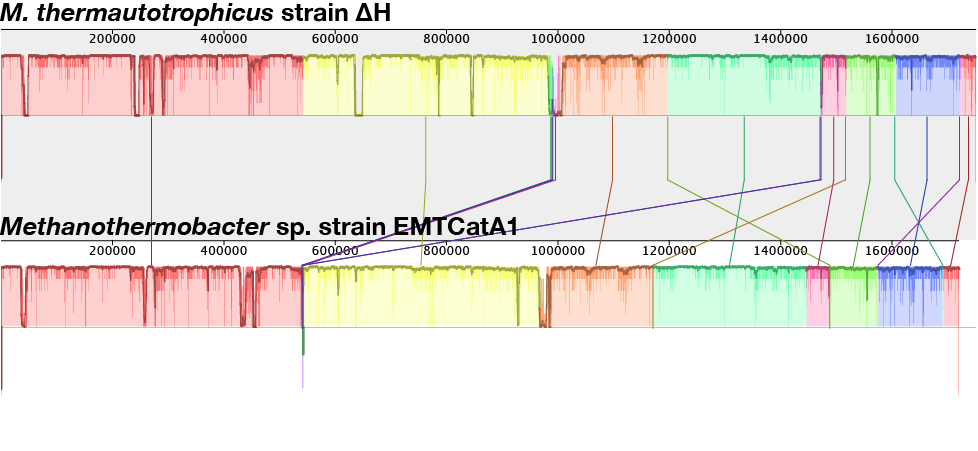


FIGURE S3: Mauve alignment of linealized genomes of *M. thermautotrophicus* strain ∆H (AE000666.1) and *Methanothermobacter* sp. strain EMTCatA1 (AP018336.1), beginning at the CDS MTH_1 and its homolog tca_00001 respectively. Blocks of genes (represented by corresponding colors) are labeled in *M. thermautotrophicus* strain ∆H.


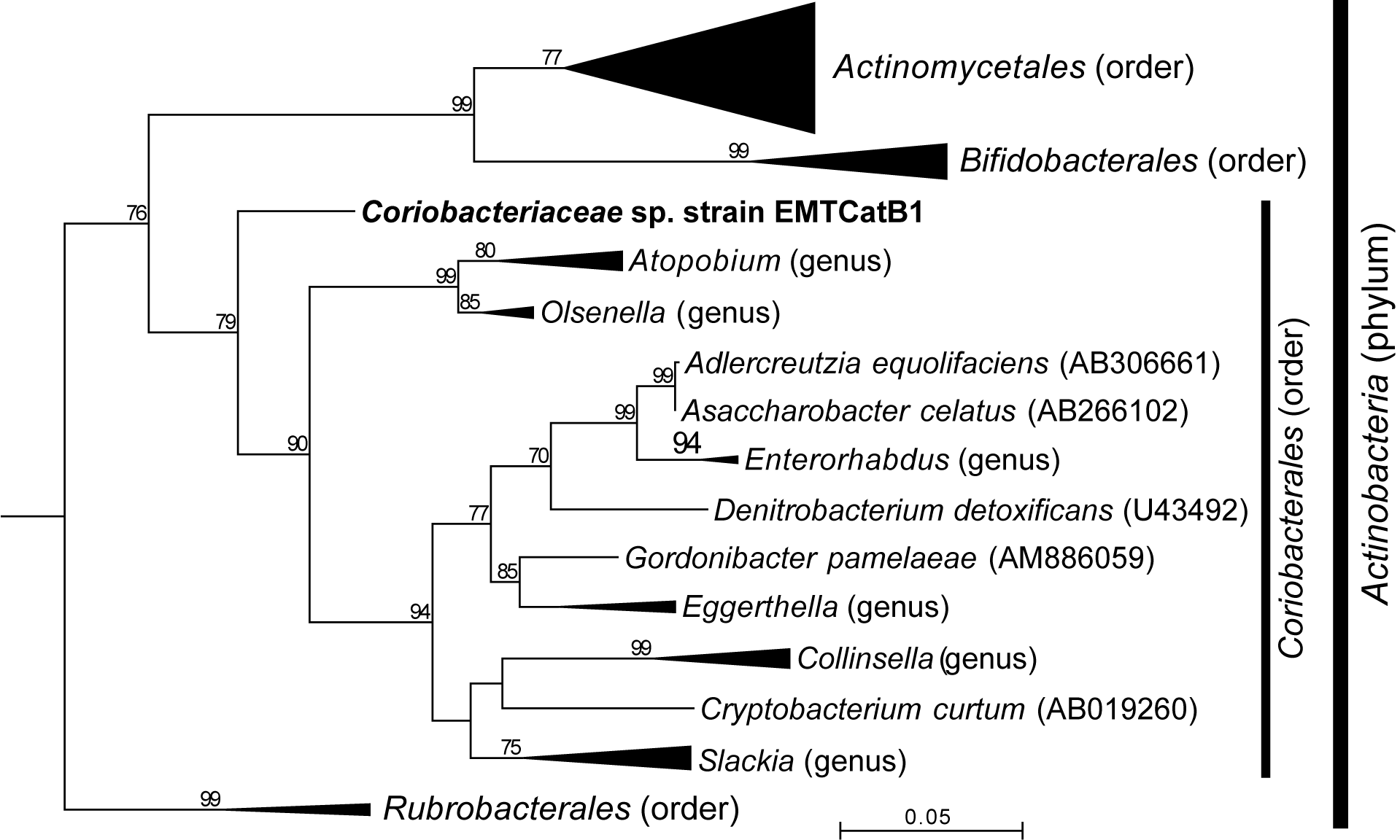


**FIGURE S4**: Phylogenetic tree illustrating relationships between *Coriobacteriaceae* sp. strain EMTCatB1 and other actinobacteria species. The tree was constructed using the neighbor-joining method. Bootstrap values (2000 replicates) of ≥50% are shown above the nodes. The scale bar shows the number of changes per nucleotide position. *Thermotoga lettingae* strain TMO (CP000812.1) served as the outgroup (not shown).


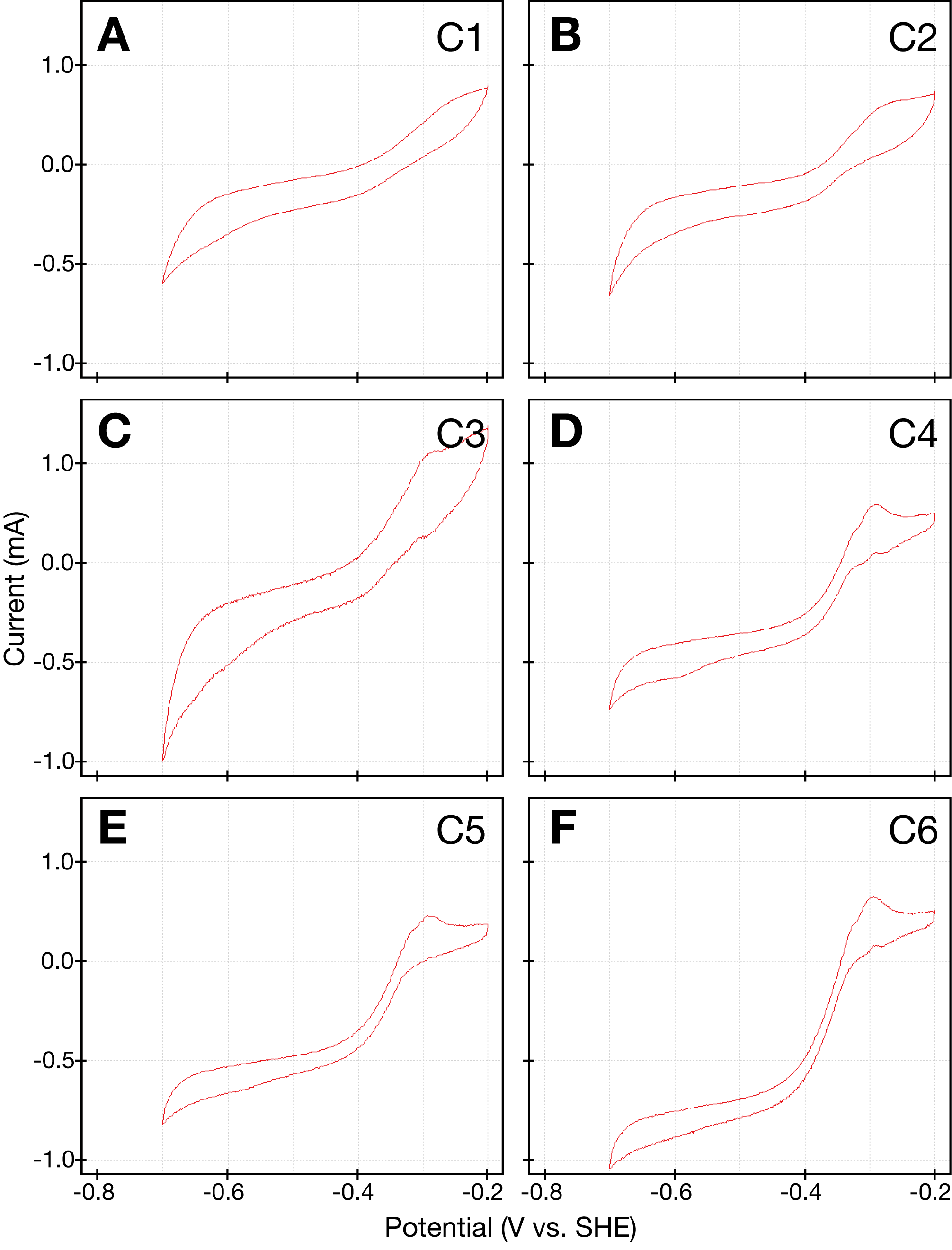


**FIGURE S5.** Cyclic voltammograms of the biocathodes of the electromethanogenic reactors C1 ~ C6 (A ~ F).


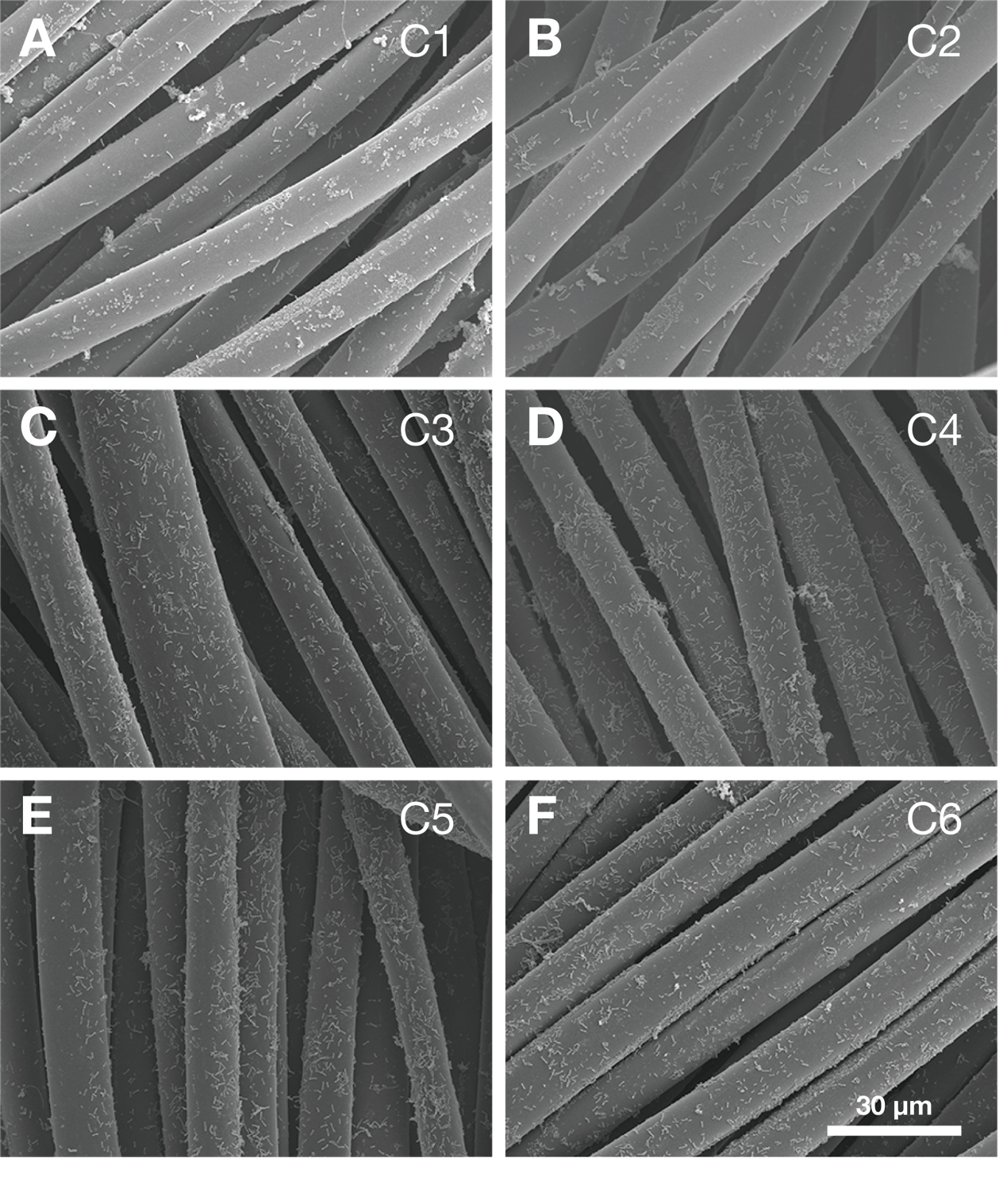


**FIGURE S6.** Representative scanning-electron micrographs of the biocathode surfaces of the electromethanogenic reactors C1 ~ C6 (A ~ F).


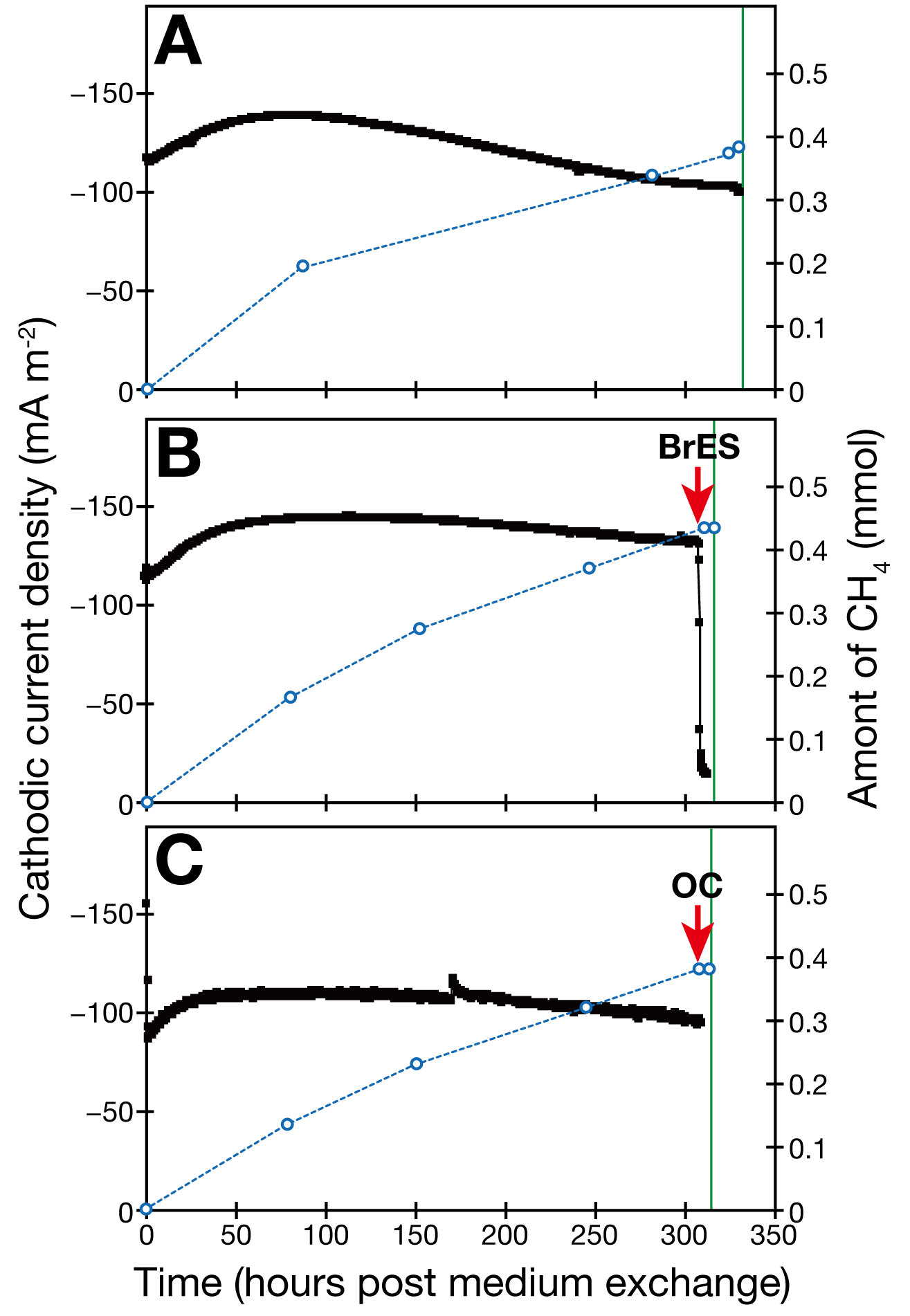


**FIGURE S7:** Current generation and CH_4_ production by the biocathodes used for transcriptome analysis under the CC (A), BrES (B) and OC (C) conditions. Each biocathode was operated at a poised potential of −0.5 V vs. SHE for three fed-batch cycles in a two-chamber reactor. Current generation (back line) and CH_4_ production (blue line) by the biocathodes at the third fed-batch cycle are shown. After approximately 2 weeks (approximately 310–330 h) following medium exchange, the biocathodes were sacrificed for RNA extraction (green lines). Red arrows indicate the addition of the methanogen inhibitor BrES (B) and the time when the circuit was opened (C), respectively. The experiment was performed in duplicate and the figure shows representative experiments.


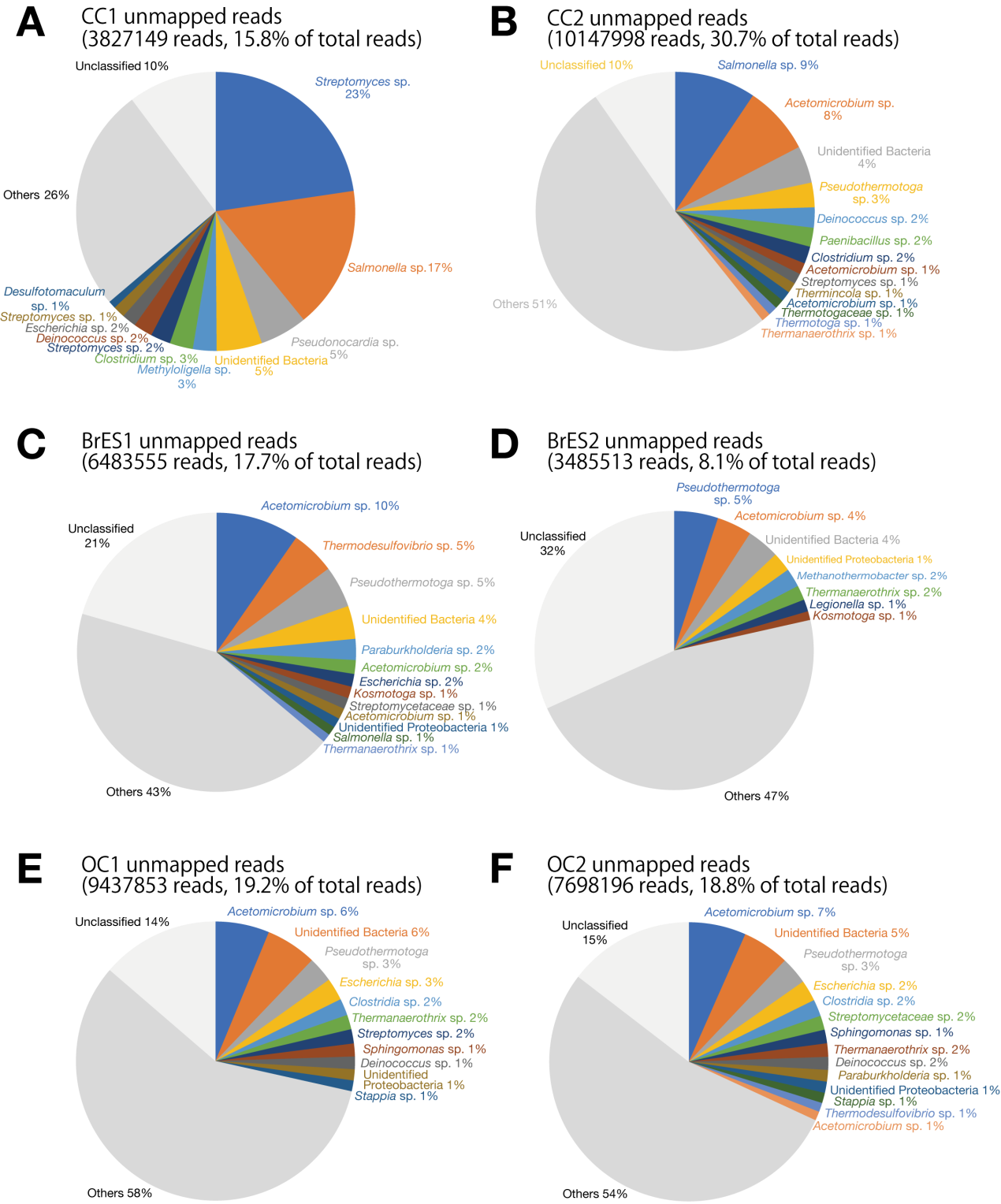


**FIGURE S8:** Taxonomic assignment of unassembled RNA-seq reads, which were not mapped onto *Methanothermobacter* sp. strain EMTCatA1 and *Coriobacteriaceae* sp. strain EMTCatB1 genomes, in metatranscriptome samples (CC1, CC2, BrES1, BrES2, OC1, and OC2) (A–F). Relative abundance of major taxonomic groups (species level) are shown. Taxa representing <1% of sequences are grouped in the ‘Others’ category.
